# Autoantibodies against myelin oligodendrocyte glycoprotein in a subgroup of patients with psychotic symptoms

**DOI:** 10.3389/fneur.2025.1593042

**Published:** 2025-07-18

**Authors:** Nikita A. van de Burgt, Laila Kulsvehagen, Marina Mané-Damas, Luc Lutz, Anne-Catherine Lecourt, Clara Monserrat, Anita M. Vinke, Cem İ. Küçükali, Shenghua Zong, Carolin Hoffmann, Emiliano González-Vioque, Celso Arango, Nicole K. Leibold, Mario Losen, Peter C. Molenaar, Erdem Tüzün, Nico J. M. van Beveren, Anna Mané, Rob P. W. Rouhl, Therese A. M. J. van Amelsvoort, for Genetic Risk and Outcome of Psychosis (GROUP) Investigators, Anne-Katrin Pröbstel, Pilar Martinez-Martinez

**Affiliations:** ^1^Department of Psychiatry and Neuropsychology, Faculty of Health and Life Sciences (FHML), Mental Health and Neuroscience Research Institute (MHeNs), Maastricht University, Maastricht, Netherlands; ^2^Mental Health and Neuroscience Research Institute (MHeNs), Maastricht University, Maastricht, Netherlands; ^3^Departments of Neurology, Biomedicine, and Clinical Research, and Research Center for Neuroimmunology and Neuroscience Basel (RC2NB), University Hospital of Basel and University of Basel, Basel, Switzerland; ^4^Institut de Neuropsiquiatria i Adiccions (INAD), Parc de Salut Mar, Barcelona, Spain; ^5^Centro de Investigación Biomédica en Red, Área de Salud Mental (CIBERSAM), Madrid, Spain; ^6^Department of Neurology, Maastricht University Medical Centre (MUMC+), Maastricht, Netherlands; ^7^Department of Neuroscience, Aziz Sancar Institute of Experimental Medicine, Istanbul University, Istanbul, Türkiye; ^8^ABC-RI, Algarve Biomedical Center Research Institute, Universidade do Algarve, Faro, Portugal; ^9^Faculdade de Medicina e Ciências Biomédicas, Universidade do Algarve, Faro, Portugal; ^10^Department of Child and Adolescent Psychiatry, Hospital General Universitario, Gregorio Marañón, School of Medicine, Universidad Complutense, IiSGM, CIBERSAM, Madrid, Spain; ^11^Department of Psychiatry, Erasmus Medical Center, Rotterdam, Netherlands; ^12^Department of Neuroscience, Erasmus Medical Center, Rotterdam, Netherlands; ^13^Academic Center of Epileptology Kempenhaeghe/MUMC+, Maastricht, Netherlands; ^14^Department of Neuroimmunology, Center of Neurology, University Hospital and University Bonn, Bonn, Germany; ^15^German Center for Neurodegenerative Diseases (DZNE), Bonn, Germany

**Keywords:** myelin oligodendrocyte glycoprotein antibody-associated disease, autoantibodies, neuroinflammation, psychiatry, mental disorders, psychosis

## Abstract

The presence of autoantibodies against myelin oligodendrocyte glycoprotein (MOG) is a hallmark of MOG antibody-associated disease (MOGAD), a recently defined demyelinating disease entity presenting with core clinical features of optic neuritis, myelitis, and acute disseminated encephalomyelitis. Although MOG antibodies have also been described in a small number of patients with other conditions, including mental disorders, their prevalence and clinical specificity in patients with isolated psychotic symptoms remain unclear. Here, we screened sera from 262 patients with at least one psychotic episode and 166 control subjects for the presence of MOG antibodies of the immunoglobulin G (IgG) isotype with a live cell-based assay. Serum reactivity to additional antigens was assessed by immunohistochemistry. Four patients, representing 1.5% of the patient cohort, and one control individual, representing. 0.6% of the healthy control cohort, were seropositive for MOG-IgG antibodies. Of the four MOG-IgG seropositive patients, three experienced visual hallucinations. Overall, MOG antibodies were detected at a low frequency in patients with psychotic episodes. While we cannot exclude the possibility of false-positive results or seroconversion due to secondary myelin damage, the association with visual hallucinations in three out of four MOG-IgG seropositive patients may point toward an underlying autoimmune etiology.

## 1 Introduction

Autoantibodies directed against brain surface proteins are rare, but when present, they generally cause neurologic symptoms, sometimes associated with psychosis. A paradigmatic example is autoimmune encephalitis caused by antibodies against the N-methyl-D-aspartate receptor (NMDAR), where after a viral prodromal phase, patients experience a wide variety of psychotic manifestations and cognitive impairment, followed by clear neurological abnormalities including seizures, movement abnormalities, autonomic instability and even coma (1–4). The clinical profiles as well as the underlying pathogenic mechanisms vary depending on the targeted antigen. Autoantibodies purely found in patients with psychotic manifestations and other mental disorders, including anxiety and depression, without associated neurological symptoms, are rare (5–7).

The presence of autoantibodies against myelin oligodendrocyte glycoprotein (MOG) is considered a hallmark of MOG antibody-associated disease (MOGAD), a recently defined demyelinating disease (8, 9) that presents with core clinical features such as optic neuritis, myelitis, and acute disseminated encephalomyelitis (8). In addition to supporting clinical and MRI features and a positive MOG-IgG test, the diagnosis of MOGAD requires exclusion of better diagnosis, including multiple sclerosis (MS) and neuromyelitis optica spectrum disorder (NMOSD).

Autoantibodies against MOG have been identified in some patients with psychosis and other psychiatric manifestations (10–12). Notably, psychotropic drugs have no or even adverse effects in a subset of patients with psychotic disorders, pointing towards a possible underlying immune etiology (13). In regard to immunomodulatory treatments in patients with psychosis, it is important to broaden the spectrum of autoantibody screening (13). In this study, we performed the first systematic screening for MOG antibodies in patients with at least one psychotic episode to investigate whether MOG antibodies play a role in the etiology of a subset of patients.

## 2 Methods

### 2.1 Participants

The study was conducted in accordance with the Helsinki Declaration.

Our cohort consisted of 262 patients with at least one psychotic episode derived from two different studies. The first study recruited patients from hospitals in the Netherlands, Spain, and Turkey [approved by the Medical Ethical Committee of Maastricht University (NL55325.068.15/METC152053, METC154126), Parc de Salut Mar (2016/6895/I) and Istanbul University (08.08.2012/1276)]. In the cohort from Netherlands and Spain, female and male individuals of at least 16 years of age (which were capable to understand the purpose and details of the study to provide written informed consent) that suffer from a psychotic disorder, defined as one or more of the following symptoms: hallucinations, delusions, thought disorders or catatonia, with an onset of disease shorter than 5 years were included. Individuals who presented with other severe brain diseases that could interfere with the neurocognitive tests, were receiving immunomodulatory treatment, or developed psychosis due to substance abuse were excluded. In the cohort from Turkey, female and male individuals of at least 18 years of age, diagnosed with schizophrenia as defined in the DSM-IV were included. Patients were excluded if they had any coexisting disease, cancer or were pregnant and if they were treated with immunosuppressive or immunomodulatory drugs.

The second study recruited patients with at least one psychotic episode [GROUP study (14)] in Amsterdam, Utrecht, Groningen, Maastricht, and Leuven (the Netherlands and Belgium) and their affiliated mental healthcare institutions under the specified inclusion and exclusion criteria (14). Available baseline serum samples from patients recruited in Amsterdam and affiliated institutions were included. Cerebrospinal fluid (CSF) from patients was analyzed when made available (*n* = 21).

For controls with a similar sex and age distribution, we used a cohort (*n* = 166) from anonymized blood donors (Sanquin Blood Supply Foundation), controls from the Spanish Psychiatric Research Network [CIBERSAM (15)] study, and controls from the GROUP study. All blood donors underwent pre-screening, including an interview with the main goal of assessing the risk of infectious diseases and risk factors (e.g., sexually transmitted diseases or foreign traveling). Additionally, individuals had to answer general questions regarding their medical history in the past 12 months, i.e., whether they had any health problems, medical appointments, surgery, or treatment. Before the initial blood donation, basic blood analytes (i.e., hemoglobin and ferritin), blood pressure, pulse and body temperature were measured. The prescreening of the blood donors included a questionnaire in which the participants were asked whether they had a chronic or severe medical condition, i.e., cancer, a cardiovascular disease, epilepsy, or a stroke. Individuals were excluded in case blood donation could have compromised their own health, in the case of severe drug abuse (i.e., use of cocaine or heroin), and if they had received an organ transplant and/or blood or blood products prior to 1980. Individuals were excluded because of a low body weight (i.e., 50 kg or less) or pregnancy. All donors tested negative for hepatitis B, C, and E, HIV, and syphilis. Psychiatric or neuropsychological functioning was not considered since systematic psychiatric and neuropsychological assessment was not available for most donors. These screening measures were conducted as part of the standard blood donor eligibility assessment and were not used for inclusion or exclusion in the current study, nor did these measures influence the interpretation of MOG-IgG seropositivity.

The controls from the CIBERSAM underwent an interview regarding their health status. Control subjects were excluded in case of a psychiatric diagnosis according to DSM-IV criteria, the presence of a severe medical condition, and current or past treatment with an antipsychotic (15). For controls of the GROUP study, individuals were excluded in case of a lifetime psychotic disorder or a first-degree family member with a lifetime psychotic disorder (14).

### 2.2 Neuropsychological assessment

Psychiatric diagnosis was established by the treating clinician based on DSM-IV criteria. The Comprehensive Assessment of Symptoms and History (CASH) was used to confirm the diagnosis in the case of patients recruited throughout the Netherlands. The severity of psychotic symptoms and global functioning were assessed using the Positive and Negative Syndrome Scale (PANSS) and Global Assessment of Functioning (GAF) score.

### 2.3 Autoantibodies against known antigens

Sera and CSF of patients and sera of controls were tested for the presence of known neuronal surface antibodies by immunohistochemistry on rat brain sections, as described (6).

### 2.4 MOG-IgG live cell-based flow cytometry assay

Sera and CSF were examined for the presence of IgG antibody reactivity against native conformational human MOG (hMOG) using a live cell-based flow cytometry assay, as described (16). In brief, sera (1:100) and CSF (1:5) were incubated with a human rhabdomyosarcoma cell line stably transfected with a pRSVneo plasmid containing full-length human MOG (247 amino acids) or the empty vector. Surface-bound MOG antibodies were detected with an IgG Fcγ fragment-specific secondary antibody. For each sample, the geometric mean channel fluorescence intensity (MFI) ratio was calculated by dividing the MFI of the MOG cell line by the MFI of the control cell line (Supplementary Figure 1A). Samples were tested up to three times on separate days and representative averages were calculated. A dilution curve (1:50 to 1:1000000) of the positively tested patients samples was performed. Data analysis was performed in FlowJo (FlowJo 10.6.2, Becton Dickinson and Company), and the cut-off for positive results was set to 3 standard deviations and a surplus of 25% above the mean of a previously reported control cohort (16).

## 3 Results

The clinical characteristics of patients and controls are presented in Table 1.

**Table 1:** Demographic and clinical information of patients, including MoG-IgG positively tested patients, and controls.

		Patients	Controls
		*n* = 262	*n* = 166
Demographics			
Age (years), mean (SD)		26.48 (8.33)	29.33 (11.63)
Sex (*n* male | *n* female)		179 | 83	85 | 81
Clinical information of included patients			
Age at disease onset (years), mean (SD)		23.52 (8.02)^a^	N/A
Number of episodes (*n*)		1.75 (2.27)^b^	N/A
Duration of illness (years), mean (SD)		2.91 (3.44)^a^	N/A
GAF, mean (SD)			
GAF score		51.36 (17.24)^c^	N/A
PANSS, mean (SD)			
Positive symptoms		12.95 (7.00)^d^	N/A
Negative symptoms		14.16 (6.76)^d^	N/A
General psychopathology		28.12 (10.20)^d^	N/A
Total score		55.45 (20.62)^d^	N/A

GAF, Global assessment of functioning; N/A, not applicable; PANSS, Positive and Negative Syndrome Scale.

a 254 patients.

b 260 patients.

c 242 patients.

d 259 patients.

### 3.1 Antibodies against MOG and brain tissue assay

**Figure 1:**
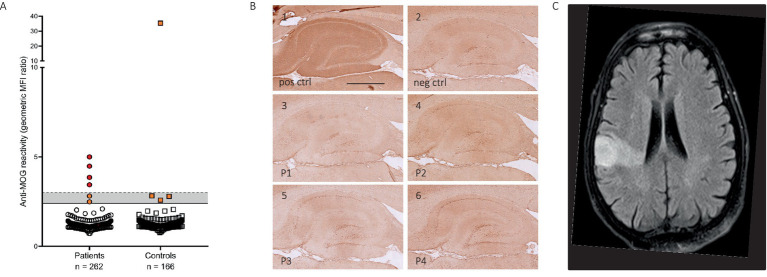
Antibodies against MOG in patients with psychosis. **(A)** MOG-IgG reactivity as the geometric mean channel fluorescence (MFI) ratio of the MOG-transfected cell line divided by the empty vector-transfected cell line. Four patients with psychosis and one control subject were seropositive for MOG-IgG, with MFI ratios varying between 3.36 and 4.29 among the seropositive patients and 35.41 for the seropositive control. **(B)** Rat brain immunohistochemistry patterns of MOG-seropositive patients. B1 represents a positive staining result from the serum of a patient with DPPX antibodies, B2 a negative staining result from serum of a non-disease control. B4-6 represents negative straining results from patients with MOG-IgG antibodies. **(C)** Magnetic resonance image (MRI) of the brain of case 4 showed a fluid-attenuated inversion recovery (FLAIR) hyperintensity in the right middle cerebral artery (MCA) territory. Scale bar = 500 μm.

Overall, four patients (1.5%, *n* = 4/262) and one control (0.6%, *n* = 1/166) were MOG-IgG seropositive (Figure 1A). Serum MOG-IgG MFI ratios are found in Supplementary Figure 1B and for each positive case in the case descriptions in Supplementary results. MOG-IgG reactivity was not detected in any of the available patients’ CSF samples (Figure 1B).

All samples (serum and CSF) were analyzed for their reactivity on rat brain tissue by immunohistochemistry (7). While some patient and control samples showed low immunoreactivity by rat brain immunohistochemistry, the MOG-IgG positive samples were negative in the assay (Figure 1B).

### 3.2 Case descriptions

Detailed clinical information of the positively tested patients is shown in Table 2 and case descriptions in Supplementary results. In short, two patients were diagnosed with schizophrenia (case 3 and 4) and the two other patients with an affective disorder (case 1 and 2). Three of the patients had a recent onset mental disorder (case 1, 2 and 3), while the other had an illness duration of 35 years with a relapsing course (case 4). Interestingly, three out of the four patients suffered from visual hallucinations, of which two patients presented these at the time of sampling (case 1 and 4) and another one had persistent visual hallucinations but not present at the time of sampling (case 3). All brain MRIs were unremarkable, except for case 4, which showed an infarct in the right middle cerebral artery (MCA) territory (Figure 1C). EEG showed slow wave activity in two out of the four patients (case 1 and 4).

**Table 2:** Demographic and clinical information of MOG-IgG positively tested patients.

Patient	Sex	Age	Prodromal symptoms	Clinical manifestations	Diagnosis	Age of onset	Number of psychotic episodes	Duration of illness (years)	Brain MRI findings	MOG-IgG positivity level
1	F	17	ASD diagnosed during childhood	Depression, hallucinations (including visual hallucinations), delusions, hypersomnia, psychomotor agitation and retardation, fatigue, cognitive decline, suicidal thoughts	Major Depressive Disorder, recurrent, severe with psychotic features	16	1	1	Normal	Clear-positive
2	F	20	Increased cannabis use, racing thoughts, megalomaniac ideas	Disorganized behavior and thoughts, insomnia, vigilance, and psychomotor restlessness	Bipolar I Disorder, most recent episode manic, severe with psychotic features	20	1	<1	Normal	Clear-positive
3	M	20	None	Hallucinations (including visual hallucinations), delusions, lack of motivation and focus, anhedonia, psychomotor retardation	Paranoid schizophrenia	16	2	4	N/A	Clear-positive
4	F	59	None	Hallucinations (including visual hallucinations), delusions, insomnia	Paranoid schizophrenia	24	11	35	Hyperintensities in the right middle cerebral artery territory	Clear-positive

ASD, Autism Spectrum Disorder; F, female; M, male; N/A, not available.

## 4 Discussion

Although the presence of autoantibodies has been described in some patients with mental disorders and psychosis (10–12), it remains unclear whether such autoantibodies play a pathogenic role in these patients or merely serve as bystander products. Moreover, there is a risk that clear positive test results in patients with a clinical spectrum of MOGAD are adulterated with false positive results in other neurological conditions. A case in point is the prevalence of MOG autoantibodies among patients with demyelinating disorders, such as MS and optic neuritis, which is estimated between 0.3–5%, compared with a prevalence of 0–1.3% among healthy individuals (17–22). It is possible that in demyelinating diseases, break-down fragments of myelin act as antigens for the formation of non-pathogenic autoantibodies, implying that this would support the hypothesis of an epiphenomenal positivity in these neurologic conditions. In this context it might be relevant to highlight that case 4 had an acute ischemic stroke in the territory of the MCA, which could have led to the release of myelin antigens.

In view of this the question arises as to whether the positivity in the four patients in our cohort (1.5% of total vs. 0.6% in the non-neurological control group) is the result of false-positive outcomes. For instance, this might be due to limitations of the assay rather than to the presence of pathogenic antibodies to MOG. It is also possible that MOG-IgG antibodies are genuinely present, but, as mentioned above, merely as bystanders without pathological effect.

All four MOG-IgG seropositive patients had normal brain MRI findings, without signs of prominent structural abnormalities, but subtle functional or immune-related changes might escape detection with conventional imaging. Therefore, future studies would benefit from including functional imaging assessment such as SPECT.

While the frequency of MOG positivity in our study is comparable to prior studies (22, 23) these results could be false positive especially since these patients do not match the clinical and radiologic syndromes for MOGAD and would thus not fulfill the recently published MOGAD diagnostic criteria (9). This is important as over-reliance on low positive antibodies and failure to fulfill diagnostic criteria may contribute to misdiagnosis with possibly harmful treatment with immunosuppressants. Additionally, MOG antibodies can occasionally co-occur with NMDAR antibodies (2, 24–26) however, this was not the case for the MOG-positive individuals we report here. Furthermore, no data on systemic inflammatory markers of thyroid- or tissue-specific autoantibodies (e.g., TPO or TGA) were available, limiting the assessment of alternative autoimmune encephalopathy syndromes such as Hashimoto’s encephalopathy. Unfortunately, it was not possible to collect longitudinal samples from the seropositive patients, measurements that could have provided additional information about the nature of the antibody signatures in these patients. Consequently, our study does not indicate that MOG antibodies play a causative role in psychosis in a subgroup of psychiatric patients. Nevertheless, it was interesting to observe that two of the four MOG-IgG seropositive patients presented with visual hallucinations at the time of sampling and another one had persistent visual hallucinations but not at the time of sampling. Visual hallucinations have also been described as initial symptoms in a patient with psychiatric symptoms in association with anti-GQ1b antibody syndrome (27) and in a rare MS case of pediatric onset (28) but also in adult onset MS (29), altogether underlining a potential link between certain psychiatric symptoms associated with distinct autoantibodies. Interestingly, visual hallucinations have previously been described in two MOG-IgG positive cases of older females, one with a rapid encephalitis like progression (30), and another one with acute onset of headache and fever, diagnosed with unilateral cerebral cortical encephalitis (31). Furthermore, one of the four pediatric MOG-IgG positive patients reported in a MOG-IgG positive UK cohort suffered from psychiatric manifestations, including hallucinations and interestingly, this patient was also positive for NMDAR antibodies (26).

Based on currently available data, including those in this study, routine screening for MOG-IgG in patients with isolated psychosis is clearly not indicated. Targeted testing, however, may be justified in the presence of atypical clinical features. For example, measurement of MOG-IgG in psychiatric patients that experience visual perceptual abnormalities to confirm or disconfirm the presence of specific serum or CSF autoantibodies. Future studies with well-defined clinical subgroups of psychotic patients, such as those with visual hallucinations, may thus help to clarify whether MOG antibodies or other autoreactive antibodies, such as NMDAR antibodies, play an underlying pathological role and contribute to psychiatric syndromes. Although our findings do not yet support the classification of a distinct autoimmune subtype, they highlight the importance of continued exploration into the potential role of immune mechanisms in subgroups of patients.

## Data Availability

The original contributions presented in the study are included in the article/Supplementary material, further inquiries can be directed to the corresponding author.

## Supplementary material

SUPPLEMENTARY FIGURE 1 **(A)** Visual representation of results from the live cell-based flow cytometry assay [signal from negative control cell line shown in grey and signal from MOG-expressing cell line shown in red (signals from positive control, secondary antibody only control, and positive and negative patient serum are shown, respectively)]. **(B)** Serum dilution curve of MOG-IgG seropositive patients. For patient samples with positive MOG-IgG result, measurements of the respective samples were repeated in a dilution series (1:50 to 1:1000000) to validate the bindings. **(C)** A T2 hyperintensity in the right middle cerebral artery (MCA) territory on magnetic resonance images (MRI) of the brain of case 4. Diffusion-weighted and apparent diffusion coefficient (ADC) are shown respectively.

## Glossary

CASH: comprehensive assessment of symptoms and history
CBA: cell-based assay
GAF: global assessment of functioning
HC: healthy control
IHC: immunohistochemistry
MS: multiple sclerosis
MOG: myelin oligodendrocyte glycoprotein
NMOSD: neuromyelitis optica spectrum disorder
PANSS: positive and negative symptom scale
