# Supplementary figures and images for: Autoantibodies against myelin oligodendrocyte glycoprotein in a subgroup of patients with psychotic symptoms
Source: Front Neurol. 2025 Jul 18;16:1593042. doi: 10.3389/fneur.2025.1593042 (PMC12316182; doi:10.3389/fneur.2025.1593042)

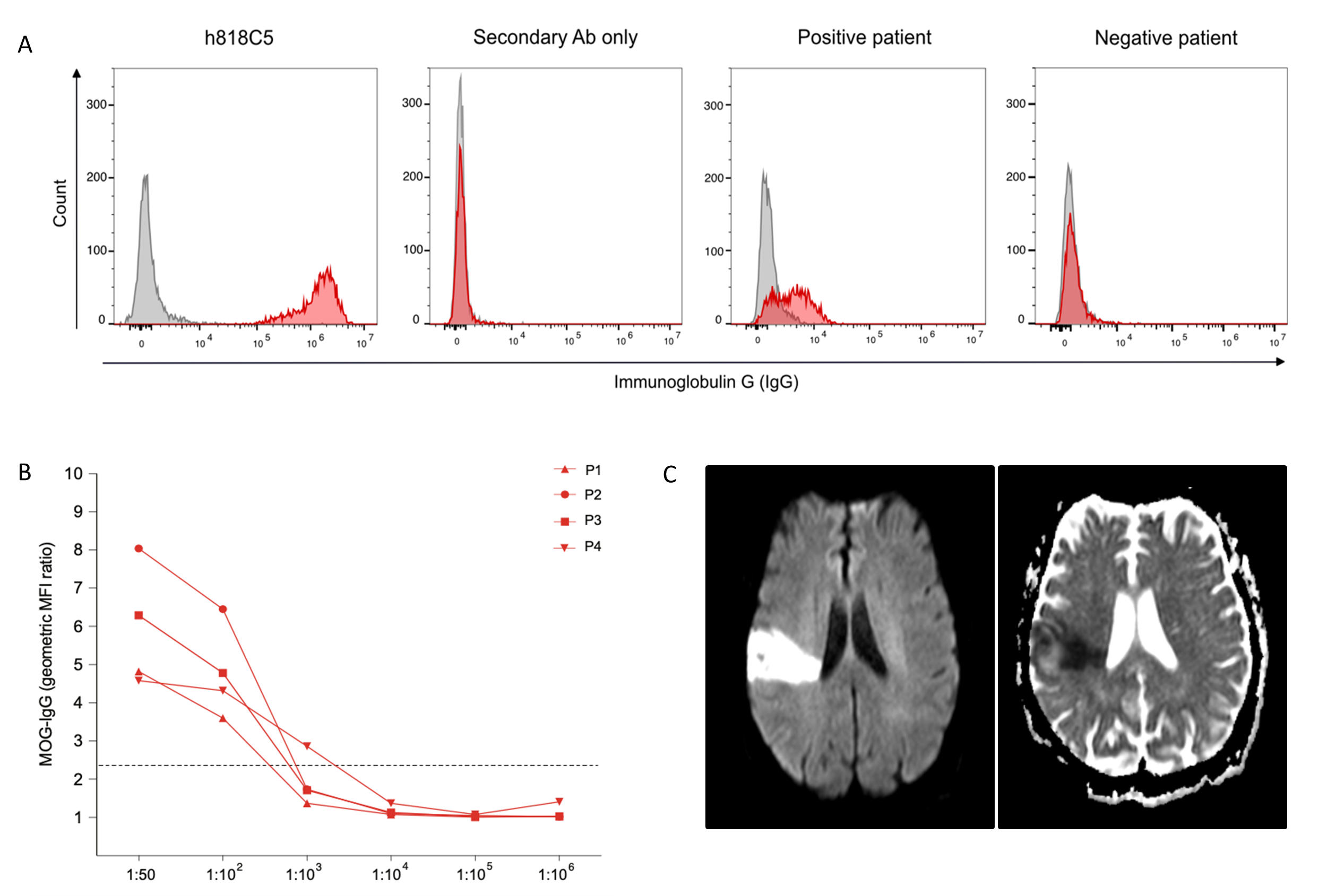

Supplement: SUPPLEMENTARY FIGURE 1 — (A) Visual representation of results from the live cell-based flow cytometry assay [signal from negative control cell line shown in grey and signal from MOG-expressing cell line shown in red (signals from positive control, secondary antibody only control, and positive and negative patient serum are shown, respectively)]. (B) Serum dilution curve of MOG-IgG seropositive patients. For patient samples with positive MOG-IgG result, measurements of the respective samples were repeated in a dilution series (1:50 to 1:1000000) to validate the bindings. (C) A T2 hyperintensity in the right middle cerebral artery (MCA) territory on magnetic resonance images (MRI) of the brain of case 4. Diffusion-weighted and apparent diffusion coefficient (ADC) are shown respectively. [file Image_1.tif]
