# Supplementary material for: Autoantibodies against myelin oligodendrocyte glycoprotein in a subgroup of patients with psychotic symptoms
Source: Front Neurol. 2025 Jul 18;16:1593042. doi: 10.3389/fneur.2025.1593042 (PMC12316182; doi:10.3389/fneur.2025.1593042)
Supplement: Supplementary file 2 [file Supplementary_file_1.docx]

**Supplementary Results**

Case descriptions

Case 1

A girl with epileptic seizures was admitted to the neurological department. Since age 15, she had been suffering from depression, followed by hallucinations, delusions, hypersomnia, psychomotor agitation and retardation, fatigue, cognitive decline, and suicidal thoughts from age 16. The depressive-like symptoms fluctuated, whereas hallucinations and delusions were present throughout. The patient used haloperidol and fluoxetine. A routine neurological examination did not reveal abnormalities other than a lateral tongue bite. She also suffered from other bodily sensations, such as electric feelings in her leg and tingling sensations in her right upper arm, hands, and lower jaw. Electroencephalograms (EEG) could not confirm epileptic activity, although slow waves in the occipital lobe on the right side of the brain could repeatedly be observed. She was diagnosed with psychogenic, non-epileptic seizures. Carbamazepine and valproic acid were prescribed daily, as well as midazolam and oxazepam when needed. At the time of inclusion, a GAF score of 40 and a total PANSS score of 54, with a positive and negative scale of 16 and 7, respectively, were detected. After being tested seropositive for MOG autoantibodies, she was referred to the neurologist for a second time. The clinical assessment and brain MRI, however, were unremarkable. Immunomodulatory treatment was not started.

Case 2

A women with insomnia, disorganization, and suspiciousness was admitted to the emergency department. Her medical history was unremarkable; however, she had a 1st-degree family history of an eating disorder. Several days after admission, she suffered from increased insomnia, mood instability, and disorganized thoughts and behavior. In the same period, she lost 5 kg of weight. During admission, her behavior remained disorganized, and she developed vigilance and psychomotor restlessness. A GAF score of 30 and a total PANSS score of 48 were detected with a positive and negative scale of 14 and 7, respectively. The psychiatric examination did not reveal insight into her psychiatric illness. Blood cell count, thyroid hormones, standard blood tests, liver enzyme levels, and brain MRI were unremarkable. The patient’s mood stabilized during treatment with olanzapine, and sleep disturbances disappeared. Seven days after admission, she was discharged. Currently, she is still stable while being treated with aripiprazole and has a good functioning daily life.

Case 3

A boy was admitted to a mental health institution because of a first psychotic episode and depressive symptoms. He experienced hallucinations, delusions, significant loss of motivation, lack of concentration, anhedonia, and psychomotor retardation. Treatment consisted of risperidone and paroxetine, and after several days, he was discharged. At the age of 18, he had another psychotic episode. He was treated with quetiapine and venlafaxine and was discharged after four months. At the time of inclusion, two years later, a total PANSS score of 40, with a positive and negative scale of 7 and 13, respectively, was detected. The patient remained stable while taking both the same antipsychotic and antidepressant.

Case 4

A woman with a 35-year history of schizophrenia was admitted to an inpatient psychiatric department with a psychotic relapse. Her mental state deteriorated after the recent death of her mother and was characterized by increased paranoia resulting in social withdrawal. At the time of admission, a total PANSS score of 112 was detected, with a positive and negative scale of 24 and 27, respectively. After the patient’s first psychotic episode, at the age of 24, she was diagnosed with schizophrenia, characterized by paranoid delusions, a thought disorder, visual hallucinations, and social withdrawal. No family history of neurological or metabolic disorders was reported. Thyroid hormones, antinuclear and antineutrophil cytoplasmic antibodies, and erythrocyte sedimentation rate were unremarkable. A retrospective medical record assessment showed that the patient had relapsed every 3-4 years with episodes characterized by prolonged, treatment-resistant events of highly disorganized and fluctuating psychosis. Treatment consisted of electroconvulsive therapy, to which she responded well. At the age of 55 years, she presented to the emergency department with another relapse and a complaint of left hemiparesis while waking. A routine neurological examination revealed anosognosia, left sensory neglect, left homonymous hemianopia, and left hemiparesis. Her brain MRI showed high-intensity signal regions on fluid-attenuated inversion recovery (FLAIR) and T2-weighted images suggestive of acute infarction in the right middle cerebral artery (MCA) territory (Figure 1C and supplemental Figure 1C). Additionally, slow waves in the right cerebral hemisphere were observed in the EEG.
