# Supplementary material for: Autoantibodies against myelin oligodendrocyte glycoprotein in a subgroup of patients with psychotic symptoms
Source: Front Neurol. 2025 Jul 18;16:1593042. doi: 10.3389/fneur.2025.1593042 (PMC12316182; doi:10.3389/fneur.2025.1593042)
Supplement: Supplementary file 3 [file Table_1.docx]

**Supplementary Material**

**Supplementary Table 1.** Immunohistochemistry scores of included patients and controls

|  |  | Patients | Controls |
| --- | --- | --- | --- |
| Score± | 0 | 220 | 156 |
|  | 1 | 32 | 4 |
|  | 2 | 9 | 4 |
|  | 3 | 1 | 2 |
|  | 4 | 0 | 0 |

0 = negative – as the negative control (non-disease human sera) 1 = borderline, 2 = weak positive, 3 = positive and 4 = strong positive - as the positive control (NMDAR or DPPX). ±All samples with IHC score 1-3 were tested negative by CBA and/or on rat hippocampal primary live neurons for the presence of known neuronal surface antigens. All MOG-IgG positive patients were tested negative by immunohistochemistry (score 0).
